# Subjective Cognitive Dysfunction in Patients With and Without Fibromyalgia: Prevalence, Predictors, Correlates, and Consequences

**DOI:** 10.7759/cureus.20351

**Published:** 2021-12-11

**Authors:** Frederick Wolfe, Johannes J Rasker, Peter ten Klooster, Winfried Häuser

**Affiliations:** 1 Research, National Data Bank for Rheumatic Diseases, Wichita, USA; 2 Internal Medicine, University of Kansas School of Medicine, Wichita, USA; 3 Behavior Management and Social Sciences, University of Twente, Enschede, NLD; 4 Rheumatology, University of Twente, Enschede, NLD; 5 Psychosomatic Medicine and Psychotherapy, Technische Universität München, Munich, DEU; 6 Internal Medicine, Klinikum Saarbrücken, Saarbrücken, DEU

**Keywords:** pain, widespread pain, somatic symptoms, fibromyalgia, fibrofog, cognitive dysfunction

## Abstract

Background: Subjective cognitive dysfunction (SCD) is common in fibromyalgia (FM), where it has been called 'fibrofog.' But its predictors and correlates are not well understood, including the extent to which SCD is present in fibromyalgia and non-fibromyalgia clinical populations. In addition, there are no studies available concerning SCD and fibromyalgia in the general population. We investigated these issues in a longitudinal rheumatic disease research databank and two cross-sectional general population studies.

Methods: 11,150 unselected patients with rheumatoid arthritis completed an assessment of FM and cognitive severity (CS) status using the full 0-3 fibromyalgia 2016 criteria assessment. In addition, CS was dummy coded as present/absent (CS+). Assessments of SCD and fibromyalgia prevalence were available in two German general population studies.

Results: Fibromyalgia was present (FM+) in 2,493 (21.7%) of clinical subjects and absent (FM-) in 9,017 (78.3%) by FM 2016 criteria. Cognitive severity was present in 1,304 (52.3%) of those with fibromyalgia and 1,009 (11.2%) of non-fibromyalgia subjects (FM-). In two general population studies, 42.0% to 52.3% of those with fibromyalgia were CS+ as were 1.4% to 5.5% of FM- subjects. Patients with CS+ had more abnormal scores for every measure of rheumatoid arthritis (RA) severity, fibromyalgia severity, and general health. The presence of CS+ was strongly related to somatic and non-somatic symptoms scores and less strongly to pain variables. The best predictor of CS+ in the clinic and the general population was the symptom severity scale (SSS), a criterion of fibromyalgia.

Conclusions: Persons with SCD have high counts of somatic and psychological symptoms. Subjective cognitive dysfunction is best predicted by a simple symptoms score, and not by pain extent scores. Although SCD is called fibrofog in patients with FM, 43.6% of CS+ cases occurred in FM- subjects. Fibromyalgia and CS are correlated but appear to be different parts of a symptom severity continuum. 'Fibrofog' as a phenomenon linked only to fibromyalgia is a misnomer because it can be identified in many non-fibromyalgia patients as well.

## Introduction

Cognitive dysfunction as it relates to patients with musculoskeletal pain is almost universally diagnosed based on self-report. We use the term subjective cognitive dysfunction (SCD), as it has been used by others [1-4], to indicate persons who report or complain of substantial “trouble” thinking or remembering. This SCD terminology includes the idea of 'fibrofog,' which is SCD in persons with fibromyalgia. “The current state of science provides no insight into the mechanistic nature of fibrofog. As no objective cognitive test is a reliable correlate of subjective dyscognition, it appears that science has so far failed to measure fibrofog," wrote Coleman et al. of SCD in fibromyalgia [3].

The complex but tenuous relation of SCD to objective mental impairment has been the subject of many studies but is not the subject of this report. Instead, we seek to understand the prevalence, predictors, and consequences of SCD in patients with and without fibromyalgia. No previous studies have evaluated SCD and fibromyalgia from this perspective.

In the report that follows, and in the context of clinical and fibromyalgia variables, we examined SCD and multiple covariates and outcomes in 11,510 patients with rheumatoid arthritis (RA) who chose one of four responses to the question, 'Do you have trouble thinking or remembering?' The options were 0: No problem; 1: Slight or mild problems, generally mild or intermittent; 2: Moderate, considerable problems, often present and/or at a moderate level; or 3: Severe, continuous, life-disturbing problems. This question is part of the series of American College of Rheumatology (ACR) related criteria for fibromyalgia [5-7], thereby permitting criteria-based diagnosis and standardized assessment of SCD.

We studied SCD in RA patients participating in a longitudinal research databank because they were evaluated with a full range of physical, social, and psychological variables. At the same time, their participation was unbiased as the presence, absence, or status of fibromyalgia-as participation was determined only by RA diagnosis. Due to the presence of pain in patients RA, they effectively are “pain” controls. We have previously shown that patients with RA are accurately diagnosed with fibromyalgia [8]. We also examined fibromyalgia and SCD data from two German population studies which included the SCD assessment [9,10] to understand if rates of SCD in persons with fibromyalgia differed according to clinical vs. population setting. Overall, in different settings and with differing fibromyalgia criteria, we examined the rates and interactions of fibromyalgia and SCD, and we investigated the implication of fibromyalgia and SCD as to disease severity, correlates, and possible causal links.

## Materials and methods

Datasets

The primary dataset for this study used data from 11,510 persons with rheumatoid arthritis (RA) participating in the semi-annual questionnaire assessment of the National Data Bank for Rheumatic Diseases (NDB) study of longitudinal outcomes. The characteristics of the NDB have been reported previously [11]. Data collection was between January 2009 and December 2014. The start date was determined by when the NDB first began collecting variables associated with fibromyalgia criteria [5-7]. In the event a study participant completed more than one semi-annual questionnaire, we selected the observation to be used by random sampling. The diagnoses of RA were made by the patient’s rheumatologist or confirmed by the patient’s physician in a small number of self-referred cases. The presence or absence of a simultaneous fibromyalgia diagnosis, if made by the referring physician, was not included with the patient referral.

We also analyzed data from two studies of the prevalence of fibromyalgia in the German general population. The first study utilized the 2011 criteria [6], including the exact (translated) wording of the criteria, as published. The second study utilized the 2016 criteria [7] and employed reworded questions [10].

Definition and abbreviations of cognitive dysfunction

Cognitive status refers to the subjective cognitive dysfunction severity assessment question outlined in the fibromyalgia criteria [5-7], as shown in Table 1. The table displays the exact language and scoring of the question. It asks if the respondent has 'trouble thinking or remembering' and includes the following scoring.

**Table 1:** The cognitive severity questionnaire The 0-3 cognitive severity questionnaire provides the basis for CS study variables, including the full cognitive severity scale (CS-F) of 0-3, the 0-1 cognitive severity positive indicator (CS+) with CS-F score ≥2, and the 0,1,2 compressed cognitive severity scale (CS-C)  with CS-F 0,1 vs. CS-F 2-3 [5-7]. CS: Cognitive severity

Score	Full Cognitive Severity Scale (CS-F)
0	No Problem
1	Slight or mild problems, generally mild or intermittent
2	Moderate, considerable problems, often present and/or at a moderate level
3	Severe, continuous, life-disturbing problems

In addition to the full cognitive severity scale (CS-F), cognitive severity positive indicator (CS+), and compressed cognitive severity scale (CS-C), we use the term SCD to refer to the subjective cognitive dysfunction concept. SCD+ is effectively equivalent to CS+.

Fibromyalgia-related variables

Widespread Pain Index (WPI)

A 0-19 score of painful sites is a measure of the extensiveness of pain [7]. Widespread pain (WSP) represents a categorical designation of body pain defined by the location and distribution of painful body sites and/or the number of painful sites. It is satisfied by the presence of pain in four or five body regions. It has no pain site requirement, but by definition requires a minimum of four painful sites [7].

Symptom Severity Score (SSS)

The SSS is a 0-12 measure of symptom severity first defined in the American College of Rheumatology (ACR) 2010 criteria and its 2011 self-report modification [7]. It includes measures of fatigue (0-3), unrefreshed sleep (0-3), cognitive difficulties (0-3), headache (0-1), pain or cramps in the lower abdomen (0-1), and depression (0-1). Cognitive difficulties (0-3) is the CS-F of this report. The SSS measures five somatic and one psychological symptom of fibromyalgia.

Adjusted SSS (ASSS)

This is the SSS score with the cognitive difficulties questions omitted. Its use allows the assessment of symptom severity and its correlates without using the cognitive difficulties score. The range of ASSS is 0-9.

Polysymptomatic Distress Scale (PSD)

The PSD, also called the fibromyalgia severity scale (0-31), is the sum of the WPI and SSS [7]. The PSD measures the magnitude and severity of fibromyalgia symptoms in those satisfying and not satisfying criteria. Fibromyalgia criteria cannot be satisfied if the PSD is <12. In the NDB dataset of this study, the best PSD cut point for FM 2016+ and negative subjects is 13.6 by the Liu test [12].

Adjusted PSD (APSD)

This is the sum of WPI and ASSS.

FM 2011

A modification of the ACR 2010 fibromyalgia criteria that allows for self-report [6].

FM 2016

A modification of FM 2011. Fibromyalgia diagnosis requires 1) WPI ≥ 7 and SSS ≥5 OR a WPI of 4-6 and an SSS score ≥9, 2) the presence of widespread pain, and 3) symptoms of at least three months duration [7].

Non-widespread pain and fibromyalgia variables

We measured the severity of pain and fatigue using 0-10 visual analog scales (VAS) in the NDB samples. The VAS measures pain intensity while WPI measures the extent of pain sites involved. Functional status was measured using the Health Assessment Questionnaire Disability Index (HAQ) [13], and overall disease activity by the patient activity score (PAS) [14]. Patients self-reported their work disability status. We also obtained each patient’s reported disability status by the US government social security pension. But social security disability does not apply after age 65. Therefore, we chose to use the self-report of work disability. Results of social security disability and self-reported disability were very similar. We measured the number of comorbid conditions (0-9) with the rheumatic disease comorbidity index [15].

Psychiatric illness now or ever was determined by respondent’s affirmative endorsement of current or ever depression, mental illness, alcohol, or drug abuse.

The full symptom count (0-37) was a count of 37 symptoms that may have been experienced by patients in the last six months. It comprised the following variables: abdominal pain, alopecia, anorexia, anxiety, asthma, bruising, constipation, depression, diarrhea, dizziness, dry eyes, dry mouth, dyspnea, epigastric pain, fatigue, fever, headache, hearing problem, heartburn, memory problem, muscle pain, muscle weakness, nausea, oral ulcers, paresthesias, photosensitivity, pleurisy, pruritus, rash, Raynaud’s phenomena, seizures, sleep problem, taste, tinnitus, urticaria, vision problem and vomiting.

The non-pain non-fibromyalgia variable symptom count (NONPNONS) of 0-26 was a shortened version of the full symptom count developed for this study. It included only variables that were not related to musculoskeletal or widespread pain, pain severity, or fibromyalgia characteristics such as alopecia, anorexia, anxiety, asthma, bruising, constipation, diarrhea, dizziness, dry eyes, dry mouth, dyspnea, fever, hearing problem, nausea, oral ulcers, paresthesias, photosensitivity, pruritus, rash, Raynaud’s phenomena, seizures, taste, tinnitus, urticaria, vision problem, and vomiting.

The Patient Health Questionnaire 15 (PHQ-15) is a measure of somatic syndrome severity. It contains 13 somatic and two psychological items. The PHQ-15 scores of 5, 10, and 15 represent cutoff points for low, medium, and high somatic symptom severity [16]. The PHQ-4 is a two-item depression and two-item anxiety screening questionnaire. Higher scores indicated more psychological distress [17].

As additional criteria, we also studied the prevalence of fibromyalgia and CS using the London fibromyalgia criteria [18] and the ACTTION-APS Pain Taxonomy (AAPT) fibromyalgia criteria [19].

Statistics

Data were analyzed using Stata (StataCorp, College Station, TX). Comparisons of CS-F groups for the study variables in the tables were performed by least square and logistic regression analyses, unadjusted for age and sex to test if there were differences between CS-F levels. Due to the very large sample size (N=11,510), essentially all statistical comparisons were statistically significant. We chose not to report p-values, given the circumstances, except to flag the single nonsignificant case. The strength of association with CS+ was assessed by correlation and receiver operating curve i.e., area under the curve (ROC AUC) analyses. Pearson correlations were used throughout. Figures were derived from logistics and ordered logistic regression analyses followed by marginal prediction using Stata’s margins and marginsplot procedures.

Ethics

Ethical approval for each database used in this study was obtained from an institutional review board (IRB) and was conducted as per the Declaration of the World Medical Association. Informed consent from human subjects was obtained as required.

German Population Studies

This first study was approved by the Institutional Ethics Review 18260 Board of the University of Leipzig (Az2-12-05032012). The second study was approved by the Institutional Ethics Review Board of the University of Leipzig (Az 145/19-ek).

National Data Bank for Rheumatic Diseases Database

This study was approved by the Via Christi IRB, Wichita, Kansas, USA (FWA00001005).

## Results

The relation of SCD to clinical and demographic variables

Of the 11,510 RA patients in this study, 20.1% reported moderate or severe SCD (CS+) as seen in Table 2. The CS-F severity was inversely associated with age. The mean age of those without SCD (CS-F=0) was 62.0 (13.0) years, and it decreased to 54.1 (12.9) in those with severe dysfunction (CS-F=3). Being CS+ was also more common in women than men (odds ratio 1.6, 95% CI, 1.4-1.8), and 78.7% of those with CS-F = 0 were women compared with 88.3% in those with CS-F = 3.

**Table 2:** The associations of the subjective cognitive dysfunction score (CS-F) with demographic, rheumatoid arthritis, and other clinical variables. CS-F: Full  cognitive severity scale, NSAIDS: Nonsteroidal anti-inflammatory drugs, VAS: Visual analog scale, HAQ: Health assessment questionnaire, ns: The difference between CS-F levels was 'not significant' at 0.05

Subjective cognitive dysfunction (CS-F)	None (0)	Mild (1)	Moderate (2)	Severe (3)	Total
N (%)	4,823 (41.9)	4,374 (38.0)	1,817 (15.8)	496 (4.3)	11,510 (100.0)
Age (years)	62.0 (13.0)	61.1 (13.4)	57.6 (13.0)	54.1 (12.9)	60.6 (13.3)
Female (%)	78.7	83.4	86.7	88.3	82.2
College graduate (%)	41.8	37.8	30.9	30.2	38.1
Married (%)	72.1	70.2	67.4	62.7	70.2
Body mass index (BMI)	28.0 (6.5)	29.0 (7.2)	30.2 (7.9)	31.3 (8.4)	28.9 (7.2)
Current smoker (%)	10.2	10.1	13.6	22.2	11.2
VAS Pain scale	2.8 (2.5)	3.9 (2.6)	5.6 (2.6)	6.5 (2.6)	3.8 (2.8)
HAQ disability index	0.7 (0.7)	1.1 (0.7)	1.4 (0.6)	1.7 (0.6)	1.0 (0.7)
Patient global	2.7 (2.2)	3.9 (2.3)	5.5 (2.3)	6.4 (2.4)	3.8 (2.5)
Patient activity scale	2.7 (2.0)	3.8 (2.1)	5.3 (1.9)	6.2 (2.0)	3.6 (2.3)
Patient joint count	6.2 (4.9)	8.7 (5.3)	11.3 (5.4)	12.6 (5.5)	8.3 (5.5)
Work disability (%)	8.6	13.8	26.6	41.9	14.8
Corticosteroids (%)	26.6	30.8	33.0	32.7	29.4
NSAIDS (%)	42.3	42.8	40.8	40.3	42.2
Biologics (%)	50.5	49.9	50.1	47.8	50.1 (ns)
Strong opioids (%)	4.0	7.0	13.1	15.9	7.0
Weak opioids (%)	17.9	24.7	34.9	35.8	23.8
Any opioid (%)	20.4	29.4	43.6	47.4	28.5
Non-opioid analgesic (%)	34.9	37.0	27.2	22.3	34.0
Comorbidity index	1.6 (1.5)	2.1 (1.6)	2.5 (1.7)	3.0 (1.9)	2.0 (1.7)

For almost all demographic and clinical variables studied, increasing CS-F scores were associated stepwise with more abnormal clinical states or changes in demographic characteristics as seen above in Table 1. These demographic factors included education, marital status, current smoking, and body mass index. The status and outcome variables are usually assessed in RA via VAS pain and global assessment scales, functional status (HAQ), overall disease activity (PAS), joint counts, disability status, and comorbidity. Treatment variables were also associated with CS status, including increased prednisone and opioid usage with increasing CS-F scores. Overall, the data in this section indicate a strong association between increasing CS-F scores and adverse outcomes and clinical status. While many of the differences across CS-F scores were moderate, some were quite large: work disability was 8.6% at CS-F = 0 and 41.9% at CS-F = 3. Similarly, opioid use increased from 20.4% to 47.4% at the same assessment points.

Fibromyalgia was present (FM+) in 2,493 (21.7%) of clinical study patients and absent (FM-) in 9,017 (78.3%) as per the 2016 criteria; and was common regardless of the definition of fibromyalgia used. Of the NDB patients, 43.0% to 52.3% of fibromyalgia cases satisfied the CS+ definition (Table 3), depending on the fibromyalgia criteria used.

**Table 3:** The intersection of fibromyalgia criteria positivity and subjective cognitive dysfunction. FM = Fibromyalgia, FM 2016 = 2016 fibromyalgia criteria, FM 2011 = 2011 fibromyalgia criteria, AAPT = ACTTION-APS Pain Taxonomy diagnostic criteria for fibromyalgia, London 6 = London 6 component criteria, German epi = 2020 German epidemiology study of fibromyalgia, NDB = National Data Bank for Rheumatic Diseases, CS+ = Cognitive severity positive indicator

Fibromyalgia Criteria	FM Prevalence %	FM (-) N	FM (-) % CS+ (N)	FM (+) N	FM (+) % CS+ (N)	CS+: Ratio FM+/FM- Cases
NDB: FM 2016 definition	21.7	9,017	11.2 (1,009)	2,493	52.3 (1,304)	1.29
NDB: AAPT definition	30.8	7,963	9.9 (788)	3,547	43.0 (1,525)	1.94
NDB: London 6 definition	26.2	8,496	11.4 (966)	3,014	44.7 (1,347)	1.39
German epi: FM 2016 definition	2.9	2,456	5.5 (135)	75	52.0 (39)	0.28
German epi: FM 2011 definition	2.3	2,393	1.4 (34)	52	42.3 (22)	0.65

**Figure 1:**
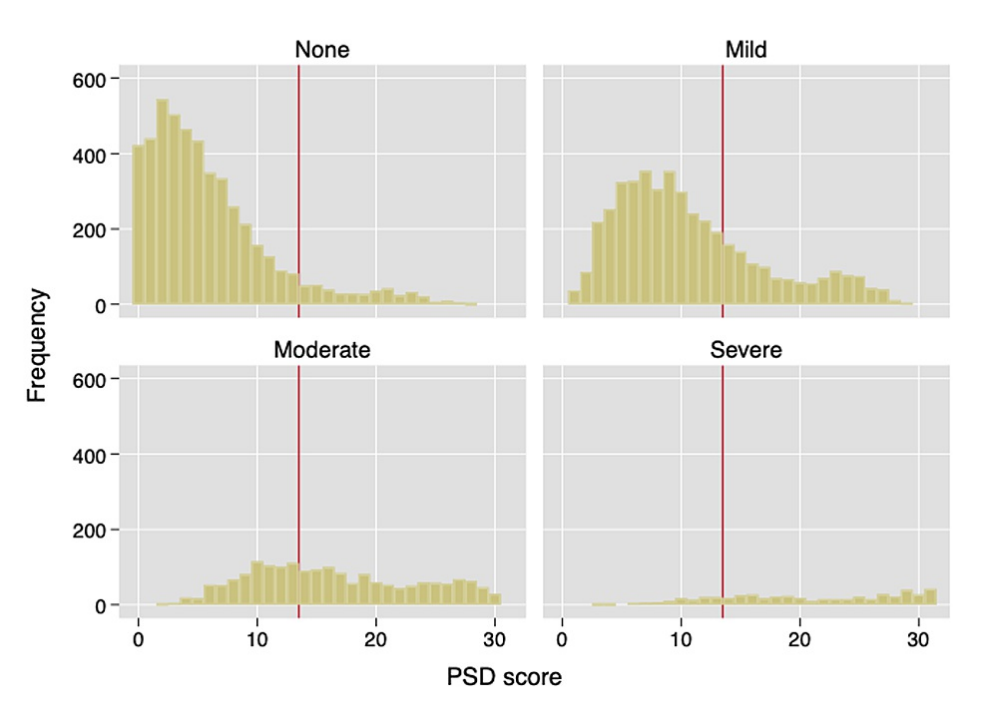
Histograms of polysymptomatic distress (PSD) scores according to full cognitive severity scores (CS-F) that are none (0), mild (1), moderate (2), and severe (3). This figure shows the distribution of PSD scores with increasing levels of CS-F. Cognitive severity groups in NDB subjects are none (n = 4,823), mild (n = 4,374), moderate (n = 1,817), and severe (n = 496). The vertical line divides histograms at PSD = 12, as no FM+ cases occur at PSD <12. Of subjects with PSD ≥12, 7,617 or 84.5% are FM+. CS-F: Full cognitive severity scale, FM+: Fibromyalgia positive, NDB = National Data Bank for Rheumatic Diseases, PSD: Polysymptomatic distress

In the general population of Germany, 42.3% to 52.0% of fibromyalgia cases satisfied the CS+ definition. Many cases of CS+, however, occur in fibromyalgia-negative subjects. The ratio of CS+ cases in fibromyalgia-positive to negative patients depends on the prevalence of fibromyalgia in the study population. In the general population, many more cases occur in fibromyalgia-negative than fibromyalgia-positive subjects. In the clinic, the ratio is reversed; for every fibromyalgia-negative case, there are 1.29 to 1.94 fibromyalgia-positive CS+ cases. From the perspective of CS+ cases in the NDB, 56.4% of those who are CS+ satisfy FM 2016 criteria and 43.6% will not satisfy FM 2016 criteria. Among CS-C negative cases, 12.9% will satisfy FM 2016 criteria. This relationship between fibromyalgia positive and negative cases and SCD status is shown graphically in Figure 1.

Correlates and predictors of SCD

Table 4 is primarily concerned with variables associated with fibromyalgia. Every measure of musculoskeletal pain, general pain, and somatic and psychological symptom severity is increased with increasing CS-F scores. 

**Table 4:** The associations of the cognitive dysfunction score with fibromyalgia-related variables. Numbers in parenthesis are standard deviations. For PHQ-4, N = 1663; for PHQ-15, N = 1,523. VAS: Visual analog scale, FM: Fibromyalgia, PHQ: Patient Health Questionnaire

Subjective cognitive dysfunction level	None	Mild	Moderate	Severe	Total
N	4,823	4,374	1,817	496	11,510
FM 2016 (+) %	4.2	22.6	53.1	68.5	21.7
Widespread pain (%)	22.2	40.6	60.2	70.0	37.3
Polysymptomatic distress (PSD) (0-31)	5.8 (5.2)	10.7 (6.2)	16.4 (6.9)	20.8 (7.3)	10.0 (7.4)
Adjusted polysymptomatic distress (PSD) (0-28)	5.8 (5.2)	8.7 (6.3)	12.5 (7.0)	15.0 (7.4)	8.4 (6.7)
Widespread pain index (WPI) (0-19)	3.7 (4.2)	6.1 (5.2)	8.9 (6.0)	10.9 (6.4)	5.7 (5.4)
Symptom severity score (SSS) (0-12)	2.1 (1.8)	4.6 (1.9)	7.5 (1.8)	9.9 (1.7)	4.3 (2.9)
Short symptom severity score (ASSS) (0-9)	2.1 (1.8)	3.6 (1.9)	5.5 (1.8)	6.9 (1.7)	3.5 (2.3)
VAS sleep scale (0-10)	2.8 (2.8)	4.3 (3.0)	6.0 (2.9)	7.0 (3.0)	4.1 (3.2)
VAS fatigue scale (0-10)	2.8 (2.6)	4.5 (2.8)	6.7 (2.4)	8.0 (2.2)	4.3 (3.1)
Count of symptoms (full) (0-36)	5.1 (4.2)	9.1 (5.5)	13.4 (6.3)	16.8 (7.1)	8.4 (6.2)
Non-pain, non-FM symptom count (0-26)	3.2 (2.9)	5.4 (3.7)	7.9 (4.5)	10.1 (5.1)	5.1 (4.1)
Currently depressed (%)	10.1	25.6	48.5	65.5	24.4
Psychiatric illness now (%)	8.7	20.2	38.4	57.1	19.8
Psychiatric illness ever (%)	32.5	51.3	67.9	81.7	47.4
Patient health questionnaire 4 (PHQ-4) (0-12)	1.0 (1.9)	2.1 (2.5)	4.4 (3.4)	7.3 (3.7)	1.3 (1.4)
Patient health questionnaire 15 (PHQ-15) (0-28)	5.9 (3.6)	8.4 (4.1)	11.9 (4.6)	14.2 (5.4)	8.4 (4.8)

**Figure 2:**
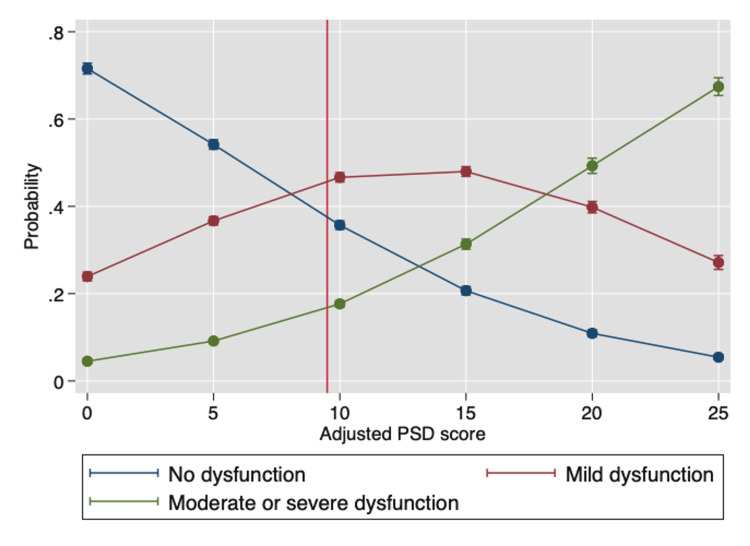
The effect of PSD score (adjusted to remove the CS variable) on the probability of scoring CS = 0 (none), CS = 1 (mild), and CS ≥2 (moderate or severe). The adjusted PSD contains the 0-19 widespread pain index (WPI) and a shortened 0-9 adjusted symptom severity scale (ASSS). The vertical line is set at 9 rather than 12 to reflect the ASSS level reduction, as the lower limit for some patients who satisfy fibromyalgia criteria in this model could be as low as 9. PSD: Polysymptomatic distress, CS: Cognitive severity, WPI: Widespread pain index, ASSS: Adjusted symptom severity scale

Figure 2 shows the effect of PSD score (adjusted to remove the CS variable contribution) on the probability of reporting CS = 0 (none), CS = 1 (mild), and CS >2 (moderate or severe). The adjusted PSD contains the WPI and a shortened symptom severity scale (SSS) that omits the CS variable. The range of PSD, therefore, is 0-28 and the PSD point below which fibromyalgia cannot be diagnosed is operationally set at 9. The figure shows how the probability of the three CS endpoints changes with increasing adjusted PSD values.

Grouping of fibromyalgia and CS categories

To understand the relationship of fibromyalgia to CS we grouped NDB patients according to their membership or non-membership in the four fibromyalgia and CS groups in Table 5. Most patients (57.6%) had neither fibromyalgia nor were CS+. Most variables demonstrated an increase in severity in the progression from FM- CS-, FM- CS+, FM+ CS-, and FM+ CS+. This can be seen clearly in clinical variables such as HAQ and disability status.

**Table 5:** Selected component and clinical outcomes according to overlapping categories of FM positivity and CS-C. Values of SSS, WPI, PSD, HAQ are mean and standard deviation (SD). FM+: Fibromyalgia positive,  FM-: Fibromyalgia negative,  CS-C: Compressed cognitive severity scale, CS+: Cognitive severity positive indicator, CS-: Cognitive severity negative indicator, SSS: Symptom severity scale, WPI: Widespread pain index, PSD: Polysymptomatic distress scale, HAQ: Health Assessment Questionnaire Disability Index

Group	N (%)	SSS (SD)	WPI (SD)	PSD (SD)	HAQ (SD)	Disabled (%)	Anxiety (%)	Ratio SSS: WPI
FM- CS-	8,008 (57.6)	2.9 (2.0)	3.7 (3.7)	6.5 (4.5)	0.8 (0.7)	8.8	13.6	1.3
FM- CS+	1,009 (8.7)	7.1 (2.0)	3.9 (2.7)	11.0 (3.5)	1.2 (0.7)	22.2	39.1	2.9
FM+ CS-	1,189 (10.3)	6.4 (1.3)	12.7 (4.3)	19.1 (4.6)	1.5 (0.6)	26.3	37.5	0.6
FM+ CS+	1,304 (11.3)	8.7 (1.8)	13.6 (4.5)	22.3 (5.2)	1.7 (0.6)	35.8	55.9	0.7
All	11,510 (!00.0)	4.3 (2.9)	5.7 (5.4)	10.0 (7.4	1.0 (0.7)	14.8	23.1	1.3

For fibromyalgia severity variables (SSS, WPI, PSD) there was a stepwise increase in severity with the different FM/CS groups, with PSD demonstrating the clearest stepwise increase. However, differences did not increase stepwise in the transition from FM- CS- to FM- CS+ for WPI, while SSS, anxiety, and self-reported disability increased disproportionately. This effect can also be seen in the changes in the ratio of SSS to WPI. These data indicate that CS status change is more correlated with symptom variable levels (SSS, anxiety, self-reported disability) and less correlated with WPI.

To further understand stronger and weaker correlates of SCD and how PSD components affect the probability of CS+, we ranked relevant predictors according to correlation with CS-F and with ROC scores for CS+ cases (Table 6). Variables with an ^a^ superscript include the actual CS score. Variables with a ^b^ superscript omit CS scores and are an appropriate study variable, as we do not want to evaluate the effect of CS when both sides of the equation contain CS. Among the variables that best predict CS-F and CS+ status is the short symptom severity score (ASSS) (CS question omitted), with a correlation of 0.608 and a receiver operating characteristic curve and area under the curve (ROC AUC) of 0.855. This variable classifies 83.7% of cases correctly. The regular symptom severity scale has a correlation of 0.773, a ROC AUC of 0.932, and a correct classification of 88.5%.

**Table 6:** Correlations of clinical and fibromyalgia variables with subjective cognitive dysfunction in all patients. ^a ^Contains cognitive severity score ^b^ Cognitive severity score omitted ROC: Receiver operating characteristic curve, AUC: Area under the curve, VAS: Visual analog scale, CS+: Cognitive severity positive indicator, FM: Fibromyalgia

Variable	Correlation with CS-F	ROC AUC for CS+
Subjective cognitive dysfunction score (CS-F)	1.000	
^a^Symptom severity score (SSS)	0.773	0.932
^b^Short symptom severity score (Short SSS)	0.608	0.855
^a^Polysymptomatic distress score	0.590	0.842
Symptom count (full)	0.547	0.808
Patient health questionnaire-4 (PHQ4) (N=1,663)	0.530	0.794
VAS fatigue score	0.519	0.812
Patient health questionnaire -5 (PHQ15) (N=1,523)	0.516	0.795
^b^Adjusted polysymptomatic distress score	0.503	0.797
Non-pain non-FM symptom score	0.476	0.768
FM 2016 criteria	0.468	0.717
Patient activity score	0.461	0.781
Patient VAS global severity	0.433	0.762
VAS sleep scale	0.409	0.739
VAS pain scale	0.399	0.749
Widespread pain index	0.391	0.724
Health assessment questionnaire (HAQ) disability score	0.382	0.730
Current depression	0.367	0.641
Patient self-reported joint count	0.365	0.710
Psychiatric illness now	0.319	0.641
Widespread pain	0.311	0.657

**Figure 3:**
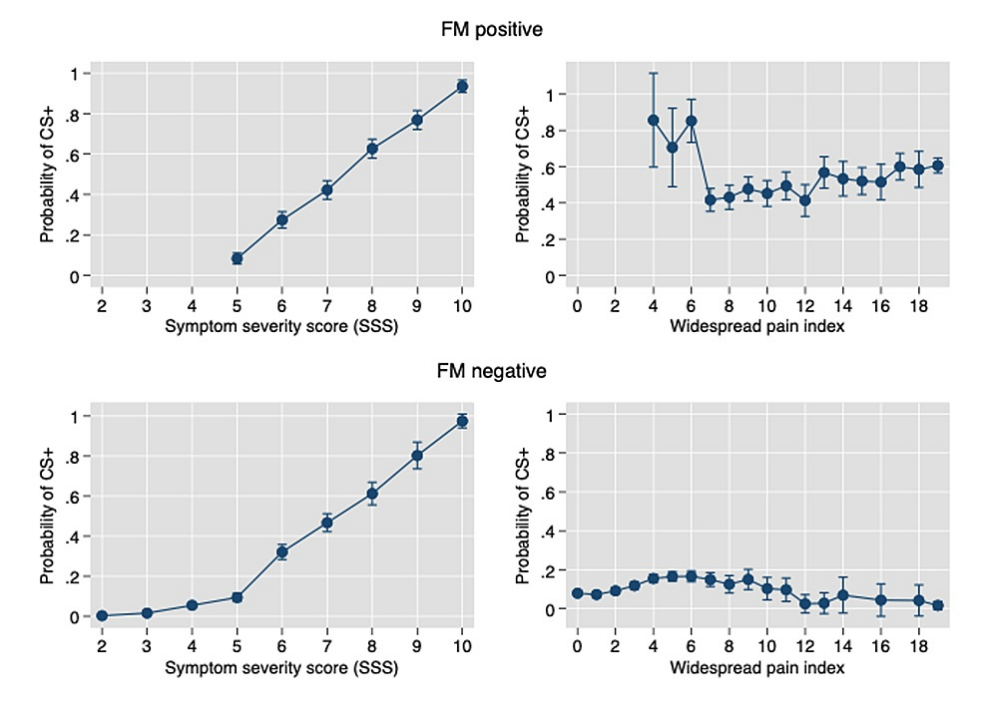
The effect of symptom severity scale (SSS) and widespread pain index (WPI) on the probability of CS+ in FM positive and FM- subjects. FM+: Fibromyalgia positive, FM-: Fibromyalgia negative, CS+: Cognitive severity positive indicator, SSS: Symptom severity scale, WPI: Widespread pain index

By contrast, the pure musculoskeletal pain variable WPI, is a much less effective predictor, with a correlation of 0.391, a ROC AUC of 0.724, and a correct classification of 79.9%. As shown in Figure 3, in FM positive and negative patients, increasing full SSS scores profoundly increases the probability of CS+. By contrast, increasing WPI scores have little effect on CS probability.

To examine more fully the relationship of somatic and psychological variables and CS+ cases, we studied SCD in the context of the NONPNONS symptom scale, as that scale contained individual symptoms generally not related to the key symptoms of the SSS scale or the WPI. The NONPNONS had a correlation of 0.476 and a ROC AUC of 0.768. It correctly classified 81.5% of cases. We regressed CS+ on each variable contained in the NONPNONS scale in Table 7. Each of the 26 variables in the scale was significantly associated with SCD+, including 26 with odds ratios > 2.

**Table 7:** The effect of subjective cognitive dysfunction (CS+) status on symptom variable positivity. CI: Confidence interval

Variable	Odds ratio (95% CI)
Nerves	4.7 (4.3-5.2)
Seizures	4.3 (2.8-6.6)
Dizziness	4.0 (3.6-4.4)
Fever	3.8 (3.2-4.5)
Vision	3.7 (3.4-4.1)
Taste problems	3.7 (3.3-4.3)
Vomiting	3.5 (3.0-4.1)
Nausea	3.4 (3.1-3.8)
Anorexia	3.3 (2.9-3.6)
Paresthesia	3.1 (2.8-3.4)
Raynaud’s phenomena	2.9 (2.5-3.2)
Mouth ulcers	2.7 (2.4-3.0)
Dry mouth	2.7 (2.4-2.9)
Asthma	2.6 (2.3-2.9)
Constipation	2.6 (2.3-2.9)
Hives	2.6 (2.1-3.1)
Photosensitivity	2.6 (2.3-2.9)
Diarrhea	2.4 (2.2-2.7)
Hair loss	2.4 (2.1-2.6)
Itching	2.4 (2.2-2.6)
Hearing problems	2.3 (2.0-2.5)
Shortness of breath	2.3 (2.1-2.6)
Rash	2.1 (1.9-2.4)
Dry eyes	1.9 (1.7-2.0)
Bruising	1.9 (1.7-2.1)
Tinnitus	1.8 (1.6-1.9)

## Discussion

In this study, we found that somatic and psychological symptoms rather than pain or pain and symptom variables were the best predictors of CS+. In fact, the addition of pain extent and location variables such as WPI and widespread pain, as in the PSD scale, did not improve CS+ prediction or identification by SSS score. The SSS score had a ROC AUC of 0.932. When the CS variable was omitted in the short SSS, the ROC AUC was 0.855-the second-best predictor. When WPI, a central component of the ACR fibromyalgia diagnostic criteria was examined [5-7], its ROC AUC was 0.724. In short, if one is looking to identify persons with CS+, symptom scales and not pain scales are the methods to use.

In the NDB data set of this study, other effective predictors of CS+ (seen in Table 6) that represented symptom scores included PSD (which included WPI), a count of 37 symptoms, VAS fatigue, the PHQ-15, and the NONPNONS score. The NONPNONS scale is of particular interest because it contained symptoms (listed in Table 6) mostly unrelated to cognition or fibromyalgia. Findings such as this suggest a strong link between reporting of many symptoms and CS+. This association has been reported previously but in much smaller and more restrictive settings [1,20-23].

Persons reporting increased CS scores have worse outcomes for all RA variables and for all fibromyalgia-related variables (Tables 4 and 5). Fibromyalgia as per the 2016 criteria is found in 53.1% and 68.5% of those with CS-F scores of 2 and 3, and 60.2% and 70.0% with widespread pain are found in those categories. Data such as these beg the question as to whether fibromyalgia is a necessary condition for CS+ or 'fibrofog.' Rather it could be argued that the increase in symptoms is the primary driver of fibromyalgia and that pain increase is correlated with symptoms. It would seem based on these data that the idea of fibromyalgia as being essentially a pain disorder might represent an oversimplification and a misunderstanding [24].

Most previous studies have approached the idea of SCD in patients with fibromyalgia, and not in chronic pain patients of differing causes. Where controls were present, they tended to be “healthy” controls. No studies used formal fibromyalgia criteria applied at the time of the study. Comprehensive clinical and fibromyalgia status variables were usually not available. Williams et al. studied 72 persons with fibromyalgia and 44 matched controls [4]. They reported that those with fibromyalgia scored significantly higher on all self-report measures of dyscognition than healthy controls, and that perceived dyscognition was most strongly associated with fatigue and mood. No studies have covered the material and questions we explored in this report. However, a number of reports have suggested an increase in symptom reporting in SCD in fibromyalgia [25-27], and our data agree with such assessments.

Recent studies with which our data agree conclude that cognitive impairment is associated with a variety of pain conditions including the “regional condition of low back pain, the widespread condition of fibromyalgia, the neuropathic condition of diabetic neuropathy” [28], and overlapping chronic pain conditions [29].

One possible limitation to our report is that we determined cognitive dysfunction with a single question that was part of the ACR 2010 criteria [5-7], but has not been validated against other questionnaires. Well validated and more comprehensive questionnaires exist [30]. However, the single item we used asks the question as it might be asked in a clinical encounter and is also part of the fibromyalgia criteria system. We believe that to assess subject dyscognition, the scale we used is appropriate. Another possible concern is the use of patients with rheumatoid arthritis as our primary data source. Getting unbiased samples and pain control is a difficult problem. The use of patients with RA overcomes the difficulty of biased samples of patients with fibromyalgia, as patients in this study were not selected for fibromyalgia characteristics or severity. We have previously shown the equivalence of fibromyalgia in RA with "primary" fibromyalgia [8].

## Conclusions

A characteristic feature of SCD appears to be high counts of somatic and psychological symptoms. In a population of pain patients, 56.4% of those who are CS+ will satisfy FM 2016 criteria and 43.6% will not satisfy FM 2016 criteria. Among CS-C negative subjects, 12.9% will satisfy FM 2016 criteria. Persons with CS+ have abnormal scores on virtually every clinical and fibromyalgia-related assessment. The SCD is best predicted by a simple symptoms score, and not by pain extent scores. Although SCD is called fibrofog in patients with FM, 43.6% of CS+ cases occurred in FM- subjects. Fibromyalgia and CS are correlated but appear to be different parts of a symptom severity continuum. 'Fibrofog' is a misnomer. We suggest that it may be more appropriate to consider both conditions as part of continuums in many diseases rather than thinking of fibromyalgia symptoms, including cognitive problems, as a discrete disorder.
